# Impact of pharmaceutical care on the quality of life of patients with Chagas disease and heart failure: randomized clinical trial

**DOI:** 10.1186/1745-6215-13-244

**Published:** 2012-12-27

**Authors:** Gilberto M Sperandio da Silva, Mayara C Chambela, Andrea S Sousa, Luiz Henrique C Sangenis, Sergio S Xavier, Andréa R Costa, Pedro Emmanuel AA Brasil, Alejandro M Hasslocher-Moreno, Roberto M Saraiva

**Affiliations:** 1 Evandro Chagas Clinical Research Institute, Oswaldo Cruz Foundation, Av. Brasil 4365, Rio de Janeiro, RJ, 21040-900, Brazil

**Keywords:** Chagas disease, Heart failure, Pharmaceutical care, Quality of life

## Abstract

**Background:**

Pharmaceutical care is the direct interaction between pharmacist and patient, in order to improve therapeutic compliance, promote adequate pharmacotherapeutic follow-up, and improve quality of life. Pharmaceutical care may be effective in reducing complications and in improving the quality of life of patients with chronic diseases, like Chagas heart disease, while bringing a positive impact on health system costs. The morbidity and mortality indexes for patients with Chagas heart disease are high, especially if this heart disease is complicated by heart failure. In this setting, we hypothesize that pharmaceutical care might be an important tool for the clinical management of these patients by improving their quality of life, as a better compliance to their treatment and the avoidance and prompt correction of drug-related problems will minimize their symptoms, improve their functional class, and decrease the number of hospital admissions. Therefore, the aim of this trial is to evaluate the contribution of pharmaceutical care to clinical treatment of patients with Chagas heart disease complicated by heart failure.

**Methods/design:**

A prospective, single-center randomized clinical trial will be conducted in patients with Chagas heart disease complicated by heart failure. A total of 88 patients will be randomly assigned into two parallel groups: an intervention group will receive standard care and pharmaceutical care, and a control group will receive only standard care. Both groups will be subjected to a follow-up period of 12 months. The primary outcome of this trial is the evaluation of quality of life, measured by the 36-item short-form and the Minnesota Living with Heart Failure Questionnaire. Secondary outcomes include drug-related problems, exercise tolerance as measured by the standard six-minute-walk test, and compliance.

**Discussion:**

Patients with Chagas heart disease complicated by heart failure under pharmaceutical care are expected to improve their quality of life, present with a lower incidence of drug-related problems, improve their functional capacity, and improve in their compliance to treatment.

**Trial registration:**

ClinicalTrials.gov Identifier: NCT01566617

## Background

Chagas disease is still a major public health problem in Latin America. About 10 million people worldwide are estimated to be infected with *Trypanosoma cruzi*, mostly in Latin America and 20 to 30% of these individuals are expected to present cardiac complications during their lifetime [1]. According to the Brazilian Ministry of Health, there are still about 2.5 million individuals living with chronic Chagas disease in Brazil and, in 2009, 4,700 deaths attributed primarily to Chagas disease were reported in Brazil [2].

Chagas disease has two distinct clinical phases, acute and chronic [3]. The majority of acute cases are clinically silent and last for 6 to 8 weeks. Only less than 10% of acute cases will present a self-limiting febrile illness, which can be aggravated by acute myocarditis. The acute phase is followed by a chronic phase, which has three forms: indeterminate, cardiac, and digestive. The indeterminate chronic form is a long-lasting asymptomatic phase, when patients present reactive serologic tests for Chagas disease but there is no evidence of damage to any organ [3-5]. However, two or more decades after infection, 20 to 30% of the patients will progress to the cardiac form of the disease, when the majority of the deaths and severe complications related to the disease occur [6-8]. Patients at the chronic cardiac phase may manifest heart failure (HF), ventricular and atrial arrhythmias, atrioventricular blocks, thromboembolism, stroke, and sudden death [8-10], with a high economic and mortality burden. Another 10 to 15% of the patients at the chronic phase will present evidence of digestive disease characterized by complaints of dysphagia or constipation and radiologic evidence of megaesophagus or megacolon [11]. An unspecified number of patients may present evidence of a mixed chronic form with evidence of both cardiac and digestive involvement [9].

Heart failure is a clinical syndrome that arises secondary to changes of cardiac structure or function that impair the ability of the left ventricle to fill or eject blood. The clinical manifestations are dyspnea, fatigue, and edema with impairment of functional capacity and quality of life of affected individuals. Heart failure is currently classified by the American Heart Association into four stages: A (patients at risk of developing HF); B (patients who have structural heart disease known to be associated with HF); C (patients with symptomatic HF); and D (patients with HF refractory to treatment) [12].

The treatment of patients with chronic Chagas heart disease is aimed at controlling symptoms and improving patients’ quality of life, by preventing cardiovascular complications, limiting disease progression, and increasing the overall survival rate of the patients. Unfortunately, there is no specific treatment against the parasite that can benefit patients at this stage, and treatment follows the guidelines issued to treat HF and arrhythmias due to complications caused by other etiologies [12].

In the proposed trial, we will focus on the contribution of pharmaceutical care to clinical treatment of patients with Chagas heart disease complicated by HF. The pharmacological treatment of patients with chronic Chagas heart disease complicated by HF includes such drugs as angiotensin-converting enzyme (ACE) inhibitors [13] or angiotensin II receptor blockers, beta blockers [14-18], aldosterone antagonists [18,19], digitalis, and loop diuretics. Many of these pharmacological strategies can prolong the life expectancy of patients with HF and are recommended in patients with Chagas heart disease complicated by HF [9]. Other drugs may be required as specific HF complications, such as atrial fibrillation, occur. For example, atrial fibrillation and ventricular arrhythmias will require drugs such as antiarrhythmics [12,18,20].

Another important aspect of HF treatment is the control of precipitating factors. Precipitating factors are any factors that can trigger an episode of decompensated HF. These include poor dietary or medical compliance, inappropriate discontinuation or reduction of HF therapy by the physician, use of medications known to worsen HF, inadequately treated hypertension, anemia, arrhythmias, and acute myocardial ischemia. Poor medical compliance and use of medications known to worsen HF, such as nonsteroidal anti-inflammatory drugs, calcium antagonists, thiazolidinediones, class I antiarrhythmic agents, and sotalol, are potentially preventable precipitating factors that can be minimized by pharmaceutical care [12,21]. Pharmaceutical care is a practice performed by the clinical pharmacist who delivers the patient’s prescription, in order to improve compliance, avoid underuse of appropriate drugs and optimize their dosage, avoid use of inappropriate drugs, and detect, prevent, and resolve drug-related problems (DRPs) [22] and improve the patient’s quality of life [24]. The value of this strategy has been demonstrated in distinct clinical scenarios [23-25]. In patients with HF, this intervention may improve patients’ symptoms, avoid hospitalizations, and, consequently, improve patients’ quality of life. In fact, small, short-term trials and observational data suggest that pharmaceutical care intervention reduces the risk of hospital admission and possibly mortality in patients with HF [26,27].

Drug-related problems are health problems linked to drug therapy that may affect expected results of patients’ therapy and their quality of life, while causing a high economic and mortality burden [28]. In the United States alone, the annual related costs of DRPs have been estimated as 177.4 billion dollars [29]. Several factors may contribute to DRPs, such as access to drug therapy, noncompliance to drug therapy, prescription duplicity, ineffectiveness of drug therapy, adverse drug reactions (ADRs), and dosage above the therapeutic range [30].

Chronic diseases, such as HF, impose a great limitation on the patients’ quality of life [31] and the success of treatment strategies for HF is measured not only by the increase in event-free time survival but also by the improvement in the quality of life of the patient [21]. Pharmaceutical care may improve a patient’s quality of life related to HF [21] and it is important to test this hypothesis in patients with Chagas heart disease complicated by HF, who, in Brazil, are mostly assisted in public outpatient services. Therefore, our aim is to evaluate the effect of pharmaceutical care, as compared with standard care, on the quality of life of patients with Chagas heart disease complicated by HF.

## Methods/design

### Study design

The proposed clinical trial will be held at a single center, the Evandro Chagas Clinical Research Institute (IPEC), which is one of the technical scientific units of the Oswaldo Cruz Foundation (Fiocruz). IPEC is a 93-year-old institution fully dedicated to clinical research in infectious diseases, including Chagas disease, among others. IPEC is a major reference center for research, care, and training on Chagas disease.

This is a double-blinded, controlled, superiority (parallel) clinical trial with balanced randomization [1:1] that will be conducted for adult volunteers with Chagas disease complicated by HF. The patients’ functional status will be classified according the New York Heart Association (NYHA) functional classification system from I to IV. All patients at our institution are treated following current guidelines for diagnosis and management of HF in adults [12].

The cardiologists from our outpatient center will refer patients with chronic Chagas disease complicated by HF to the pharmacist. The pharmacist will explain the study in detail and obtain the patients’ informed consent. All included patients will undergo a screening phase, which includes a review of the patient’s medical records and an interview for assessment of eligibility. All eligible patients will complete quality-of-life questionnaires and take a six-minute-walk test after inclusion. After the screening phase, patients will be randomly assigned to one of two groups: an intervention group, who will receive standard care plus pharmaceutical care, and a control group, who will receive only standard care.

**Figure 1:**
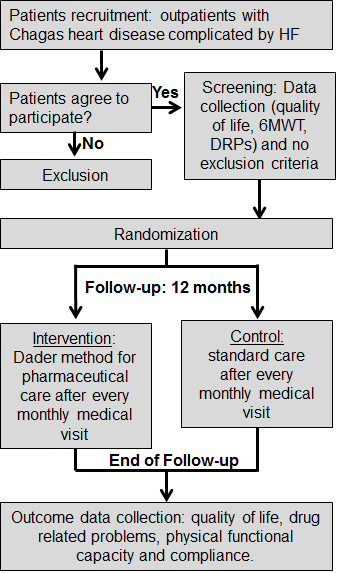
**General overview of study protocol.** 6MWT, six-minute-walk test; DRPs, drug-related problems; HF, heart failure.

Each patient will be followed for 12 months. All patients from both groups will take part in medical consultations every month. After each medical consultation, a pharmacist blinded to the patient’s assignment will interview all patients, to identify compliance to treatment and any DRPs. After this, all patients will interact with the clinical pharmacist. Those randomized to the control group will receive all prescription medications, while those patients randomized to the intervention group will not only receive all prescription medications but will also undergo pharmaceutical care, to solve DRPs, confirm, and reinforce their compliance to the medical prescription. Whenever the pharmacist identifies a DRP in the intervention group, he will interact with the physician, to solve the DRP. All patients will take part in a pharmaceutical consultation at the end of the follow-up, to identify DRPs, complete quality-of-life questionnaires, and perform six-minute-walk tests. The pharmacists who will deliver the initial evaluation before randomization, the monthly evaluations of DRPs and compliance, and the final evaluation at the end of the follow-up period will be blinded to the patients’ assignment (Figure 1).

### Patients: inclusion and exclusion criteria

Inclusion criteria are adults (>18 years old), men and women, with chronic Chagas disease diagnosed by two distinct Chagas serology tests (indirect immunofluorescence and ELISA), and complicated by HF. For the purpose of this study, HF diagnosis will be based on current or past symptoms compatible with HF and associated with underlying structural heart disease and left-ventricle ejection fraction under 55%, as measured by an echocardiogram. Exclusion criteria are: any comorbidities that significantly affect cardiac performance, such as coronary artery disease, moderate or severe heart valvular disease, left-ventricle hypertrophy, or congenital heart disease; comorbidities that limit survival, such as malignant tumors or HIV; failure to obtain informed consent; inability to perform six-minute-walk test; significant cognitive impairment; pregnancy; concomitant participation in other intervention trials; or NYHA functional class IV.

### Interventions: pharmaceutical care vs. standard care

The pharmaceutical care applied in this trial will be based on Dáder methodology. The Dáder method for pharmaceutical care is a systematic process developed by the Research Group of Pharmaceutical Care at the University of Granada, Spain [32]. This method includes patient education about cardiovascular drugs, patient counseling about lifestyle modifications, assessment of drug compliance, identification of underuse of appropriate drugs, optimization of drug dosage, and identification of the use of inappropriate drugs, to enable the pharmacist to deliver treatment recommendations to physicians and contribute to relieving DRPs [32] and improved quality of life [21,23].

Those patients randomized to standard care will receive all prescription medications from the pharmacist and basic instruction on how to use them.

### Outcomes

The primary endpoint of this trial will be an increase in the quality of life. Heart disease may impose important limitations on patients’ daily activities that often affect their functional and general health status [21]. Quality of life will be evaluated using the 36-item short-form (SF-36) [33] and the Minnesota Living with HF Questionnaire (MLHFQ) adapted by Carvalho *et al*. [34]. The SF-36 will be measured in all eight subscales: physical functioning, physical role, bodily pain, general health, vitality, social functioning, emotional role, and mental health. The two summary measures are the Physical Component Summary and the Mental Component Summary score. Items scores related to each subscale are coded, summed, and scaled from 0 (worst possible health state) to 100 (best possible health state) [23,33,35,36]. The MLHFQ is one of the most widely used questionnaires to evaluate quality of life of patients with HF. It is made up of 21 items that cover HF-related physical, psychological, and social impairments. The patient’s perception of such impairment is assessed on a scale ranging from ‘none’ (a score of 0) to ‘very much’ (5). The total MLHFQ score is obtained by adding the scores for all 21 items (range, 0 to 105), where a higher score indicates a worse quality of life [37].

The secondary endpoints evaluated will be incidence and types of DRPs, physical functional capacity, and compliance. Drug-related problems will be identified case by case, based on the classification of the Brazilian Pharmaceutical Care Consensus [38]. They will be classified by evaluating three distinct criteria of pharmacotherapy: indication, effectiveness, and safety [30,38]. Among DRPs, suspected ADRs will be classified according to Naranjo’s algorithm [39]. All identified DRPs will be reported in patients’ medical records. Another secondary endpoint will be physical functional capacity. All patients will have their functional capacity measured by the six-minute-walk test. The test will be performed at the beginning of protocol and after 12 months of follow-up. The test will be conducted on three consecutive days, at the same time and followed by the same evaluator. It will take place along a closed corridor, 33 m long. Walk tests will be performed at least 2 hours after patients’ meals. Patients will be instructed by a cardiologist or designed professional to walk as much as possible for 6 minutes. Patients will be oriented to adjust their walking speed between relatively easy and slightly tiring. The test will measure the maximum distance each patient can walk in the corridor [40-42]. Patients’ compliance to medical treatment will be measured using self-reports of medication adherence on the Morisky scale [43]. This simple four-question survey assesses the likelihood that patients take their medications as prescribed. The questions will be: ‘Do you forget to take your medications? Are you careless about the time at which you take your medications? Do you stop taking your medications when you feel better? Do you stop taking your medications when you feel worse?’ Each ‘yes’ answer is given a score of 1, and each ‘no’ a score of 0. Those patients scoring 0 will be considered adherent, and those scoring 1 to 4 will be considered nonadherent [43].

### Ethical issues

This study was approved by our local Committee for Ethics in Research (IRB) under the number 0034.0.009.000-11 and is registered at ClinicalTrials.gov under number NCT01566617 [44]. The IPEC IRB membership includes multidisciplinary representation as well as community representation. It is registered at the National Committee for Ethics in Research-MOH [45] and operates according to the Brazilian Ethics in Human Research Regulations.

#### Protection against risk

The informed consent process will be conducted according to good clinical practices. The potential study patients will sign an informed consent form after reading it, receiving adequate explanations, and understanding the study objectives and procedures. Illiterate patients may bring in relatives or friends to read for them, testify, and sign the consent form: if this is not possible, we can enroll a patient, and a witness will sign the consent form. The formal informed consent process will be accomplished and documented before any other study-related procedures. Adequate time will be allowed for the patient to ask questions. All aspects of the study will be explained to the patients, including, but not limited to, potential risks and benefits, and the amount of time required from the patient, including the number of study visits expected. All patients will be informed that participation is voluntary, that refusal to participate will involve no penalty or loss of benefits to which the patient is otherwise entitled, and that the patient may discontinue participation at any time without penalty or loss of benefits to which the patient is otherwise entitled.

The investigators will see patients at regular intervals. Patients who miss a scheduled study visit will be contacted by telephone to schedule a new visit. Case report forms will identify patients only by the study number. All records identifying patients by name and information linking patients’ names with study ID numbers will be kept in locked rooms in locked cabinets and will be accessible only to study personnel. We will provide patients with the names and telephone numbers of the principal investigator and sub-investigators, and they will be advised that they are allowed to make reverse-charge telephone calls. Information regarding the patient’s medical history will be obtained with the patient’s consent. Regarding this proposal, the procedures are considered to offer minimal risks for the patients. The patients may derive direct benefits derived from research and the knowledge acquired may potentially improve the health care of other patients suffering from Chagas disease. Therefore, the benefits of this research will fall into two major categories: benefits to patients and benefits to society. Patients will not receive any kind of remuneration, but will be reimbursed for travel expenses and meals.

#### Data and safety monitoring plan

The IPEC IRB will reevaluate the research project at intervals appropriate to the degree of risk but not less than once a year. Periodic review of the research activity is necessary to determine whether the risk/benefit ratio has shifted, whether there are unanticipated findings involving risks to patients, and whether any new information regarding the risks and benefits should be provided to patients. At the time of initial review, the IPEC IRB will determine whether an independent data and safety monitoring board or committee will be required, and should also set a date for reevaluating the research project. In light of the risk/benefit ratio reassessment, the IRB may require that the research be modified or halted altogether. Alternatively, special precautions or criteria for inclusion may be relaxed. The principal investigator and study team will keep the IPEC IRB informed of significant findings that may affect the risk/benefit ratio. The investigators will inform patients of any important new information that might affect their willingness to continue participating in the research.

### Data collection

At the screening phase, data regarding quality of life, six-minute-walk test results, DRPs, and clinical and sociodemographic data will be collected. Clinical and sociodemographicdata will include age, sex, functional class, heart rate, blood pressure, weight, Chagas serological test results, biochemical data (urea, creatinine, bilirubin, aspartate aminotransferase, alanine aminotransferase, and alkaline phosphatase levels), and comorbidities (hypertension, diabetes, and asthma), as well as alcoholism, smoking, and prescription drugs.

Data about DRPs will be collected at least once a month. At the end of the study follow-up, DRPs, six-minute-walk test results and MLHFQ and SF-36 scores will be collected again.

### Statistical methods

The EpiData [46] and Statistical Package for the Social Sciences [47] applications will be used for data entry and analysis, respectively. The statistical calculations will be performed on an intention-to-treat basis, where patients will be analyzed as initially randomized. Continuous variables will be expressed as mean ± standard deviation. The Kolmogorov-Smirnov test will be used to test sample distribution. The analysis strategy will be based on differences of MLHFQ and SF-36 scores, DRPs outcome, and six-minute-walk test results between the control and intervention groups. For quality-of-life questionnaires and the six-minute-walk test, the Student’s *t* test will be used if sample distribution proves to be normal. Otherwise, the Wilcoxon rank-sum test will be used. For DRPs and compliance, cumulative incidence and relative risks will be estimated with their respective 95% confidence intervals, and will be used to test differences. The null hypothesis will be rejected at *P <* 0.05.

### Sample size calculation

Sample size calculation was based on previous work of the literature that evaluated pharmaceutical care and quality of life in patients with HF [21]. We estimated that 40 patients per group would be needed to detect a difference in quality of life on SF-36 of 10.3 points, allowing a standard deviation of up to 10.9 points, one-sided 1% significance, and 95% power. As we expect loss during follow-up of at least 10% of the study participants, 88 participants (44 per group) will be required (Table 1).

**Table 1:** Sample size calculation according to scenarios of benefit in terms of quality of life for patients

**Parameter**	**Groups**		**Sample calculation**
	**Control group**	**Intervention group**	
Quality of life, mean (standard deviation)	52.8 (10.9)	63.1 (10.1)	
Significance level	1.0%	1.0%	
Power of test	95.0%	95.0%	
*N*	40 patients	40 patients	
*N* + 10% (in each group)	44 patients	44 patients	
Ratio	1:1	1:1	
Total	44	44	88

### Randomization and concealment

A computer-generated random list of allocation, to either pharmaceutical care or standard care, will be generated, balancing 1:1. This assignment will occur within randomly ordered blocks of size four, six, or eight.

The allocation sequence will be concealed from the researchers enrolling and assessing participants, in sequentially numbered opaque, sealed, and stapled envelopes. To prevent subversion of the allocation sequence, the name and date of birth of the participant will be written on the envelope before the envelope is opened. The information in the envelope will be transferred to the allocation card inside the envelope. Corresponding envelopes will be opened only after enrolled participants have completed all baseline assessments and at the time of allocation. These procedures will be carried out by a professional without access to baseline evaluation results.

### Blinding

The pharmacists who will carry out the initial evaluation before randomization, monthly evaluations of DRPs, and compliance, and the final evaluation of the health care questionnaires after the end of the patient follow-up will be blinded to the patient’s assigned group. Physicians conducting the six-minute-walk test will also be blinded to group assignment.

## Discussion

Pharmaceutical care in the treatment of patients with HF has been associated with significant reductions in the risk of all-cause hospitalizations and HF hospitalizations [21,26,27], and with an improvement of exercise tolerance and quality of life [21]. Those improvements were related to a higher compliance to prescribed medications [21] and higher ACE inhibitor doses [27] in the group of patients who received pharmaceutical care. On the other hand, Lowrie *et al*. [48] reported no improvement in the composite endpoint of death from any cause or hospital admission for worsening HF in patients with left-ventricle systolic dysfunction when submitted to pharmacist intervention. However, in this study, the training of the pharmacists was limited to brief tuition and the improvements in the prescription of outcome-modifying medications were, at most, modest.

The present proposed trial will be the first one to evaluate pharmaceutical care conducted in patients with HF due to Chagas disease. The population of patients with Chagas disease are a group of underprivileged individuals with limited access to health care services and with a high proportion of illiterate individuals [49]. Therefore, we anticipate that pharmaceutical care intervention in this group of individuals will increase the rate of compliance to prescribed medications, while helping to improve the achievement of maximum tolerated dose of outcome-modifying medications and mitigating DRPs. In consequence, we expect that the intervention group will experience an improvement in the quality of life and in exercise tolerance. We expect that the improvement attained in quality of life, as measured by MLHFQ and SF-36 scores, at the end of 12 months of follow-up will be of at least ten points. Recently, Kato and colleagues have also demonstrated that a low MLHFQ score is associated with increased risk of all-cause death as well as the combined endpoint of cardiac death or hospitalization for HF in patients with HF [37], which reinforces the value of the quality of life score used in this proposed trial.

The confirmation of the expected results in this trial will be the basis for future larger studies addressing the influence of pharmaceutical care on mortality outcomes of patients with Chagas heart disease complicated by HF.

### Trial status

This study is currently recruiting patients.

## Abbreviations

ACE: Angiotensin-converting enzyme; ADR: Adverse drug reactions; DRP: Drug-related problems; ELISA: enzyme-linked immunosorbent assay; HF: Heart failure; MLHFQ: Minnesota Living with HF Questionnaire; NYHA: New York Heart Association.

## Competing interests

The authors declare that they have no competing interests.

## Authors’ contributions

GMSS and RMS conceived the study. GMSS, AMHM, PEAAB, and RMS participated in the study design. MCC, ASS, LHCS, SSX, and RMS will recruit, select, and collect clinical data of the patients. MCC and GMSS will randomize patients into the two arms of the protocol, deliver the proposed intervention, and collect data. PEAAB, GMSS, and RMS will perform statistical analysis. GMSS is the study coordinator and RMS is the principal investigator. GMSS, ARC, PEAAB, AMHM, and RMS helped to draft the manuscript. All authors read and approved the final manuscript.
